# Adolescent Eating Disorder Day Programme Treatment Models and Outcomes: A Systematic Scoping Review

**DOI:** 10.3389/fpsyt.2021.652604

**Published:** 2021-04-29

**Authors:** Julian Baudinet, Mima Simic

**Affiliations:** ^1^Maudsley Centre for Child and Adolescent Eating Disorders (MCCAED), Maudsley Hospital, London, United Kingdom; ^2^Department of Psychological Medicine, King's College London, Institute of Psychiatry, Psychology & Neuroscience (IoPPN), London, United Kingdom

**Keywords:** adolescant, young adult, day program, partial hospitalization program, intensive outpatient program, intensive treatment program

## Abstract

**Background:** Adolescent eating disorder day programmes (DP), or partial hospitalization programs, are becoming increasingly widespread worldwide. They typically function as an alternative to inpatient care and/or a step up or down in treatment intensity. There has been an increase in the number of publications within the last 5 years investigating DP outcomes. While there are now numerous programmes operating internationally, there is large variability in the content, structure and theoretical underpinnings of each programme. This makes it difficult to compare programme outcomes, and the impact the therapeutic model may have.

**Aims:** To review existing literature on adolescent eating disorder DP treatment models and outcomes.

**Methods:** A systematic scoping review was conducted. Four databases (PsychInfo, EMBASE, Medline, CENTRAL) were searched for relevant peer-reviewed journal articles and book chapters investigating adolescent eating disorder DPs that function as alternatives to inpatient treatment. No restrictions on study methodology were imposed. Studies were first mapped by location, study characteristics and day programme treatment characteristics, then narratively synthesized.

**Results:** Forty nine studies were included in this review. All used a quantitative methodology. One study also included qualitative methods. The majority of studies included describe DPs in the USA (69%). Seventy-six percent of the studies described DPs that operate 5-days per week and most (57%) either only admit or only report on outcomes for restrictive eating disorders. Two-thirds (69%) reported on DPs that had a family focused treatment model, the remainder had a more integrated treatment model informed mostly by individual psychotherapeutic models. Generally, DP treatment is associated with weight gain and improvements in eating disorder and comorbid psychopathology. The studies that include follow-up data (27%) reveal improvements are usually maintained from 3 months to 2 years post-treatment. Early weight gain, early psychological change and early therapeutic alliance are associated with improved end of treatment outcomes. Findings regarding other potential predictors of outcome are mixed.

**Conclusions:** Current evidence suggests day programmes are an effective alternative to inpatient treatment that lead to sustained improvements. DPs tend to either be young-person-only with a family-focused treatment model or all age with a more integrative model. Controlled, empirical investigations into the impact of the therapeutic model on outcomes are needed, as are investigations into treatment mechanisms and the individual and parent experience of day programme treatment.

## Introduction

In this article, we provide a review of adolescent eating disorders day programmes (DP), focusing on theory, structure, process and outcomes. There have been significant advances in the field of adolescent eating disorder treatment over the past 50 years. Family therapy has emerged as the current first-line recommended treatment (1), with individual and multi-family therapy also demonstrating promise (2–7). Despite these advances and the increase in treatment options, full remission rates at the end of treatment remain modest for both anorexia nervosa (20–50%) and bulimia nervosa (~40%) (8). Historically, inpatient treatment was considered for this group, however, outcomes following hospitalization are mixed (9, 10) and the benefit of inpatient care beyond medical stabilization disputed (11–13).

A range of higher levels of care are now emerging as alternatives to inpatient treatment (14, 15). These programmes aim to reduce the need for inpatient admissions and better meet the needs of this group of young people and their families (16, 17). These programmes go by several different names, including DPs, partial hospitalization programs, intensive treatment programs, etc., but share some key similarities. They all offer increased treatment intensity relative to outpatient treatment, include supervised meal support, revolve around a group-based therapeutic programme and offer treatment multiple times per week for several hours per day. Some programmes specify different levels of intensity within the one programme, with young people and their families moving between them based on need and stage of treatment. When multiple levels exist, the higher level of care is typically referred to as a partial hospitalization program, whereas the lower level of care is referred to as an intensive outpatient program.

For the purposes of this review, the term DP is used to refer to any treatment programme that acts as an alternative to inpatient treatment where the young person does not stay overnight at the treatment facility (as per inpatient). Studies investigating intensive outpatient treatments only (half-days and <5 days per week, or not positioned as alternatives to inpatient treatment) or adjunctive multi-family therapy groups are not included in this review. Programs that report outcomes for combined inpatient and DP treatment are also excluded [e.g., (18–21)] as they do not typically function as alternatives to inpatient treatment. Rather they often act as step-down transition programmes between inpatient units and the community and typically aim to reduce admission lengths and readmission rates. Furthermore, outcomes for the specific DP component are also rarely reported on, making it difficult to ascertain its unique contribution.

DPs are generally considered to be preferable to inpatient treatment as they are less costly and attendees can stay connected to their family, peers and lives more generally during treatment (22, 23). Staying connected to day-to-day life is important for several reasons. Firstly, new skills developed can be immediately applied to real-life situations. Secondly, there is greater opportunity to access and build supports in the home and social environment (23). All of this can be difficult during inpatient or residential treatment, where the young person is in the facility 24-h per day and may be quite far geographically from home, family, peers and school. This is important as eating disorders may disrupt psychosocial functioning and are associated with altered patterns of responding to interpersonal stress (24, 25). Without exposure to the challenges of everyday life, the transition from hospital back to home can be difficult and may increase the risk of relapse.

Evidence is now emerging that DPs support physical and psychological improvements for young people with eating disorders and have similar outcomes compared to inpatient care (22, 26). Nevertheless, beyond sharing an increase in treatment intensity, no two DPs are identical. They vary substantially in treatment length, amount of treatment offered per day/week, the model(s) of treatment offered, the population treated and programme aims (23, 27). This can make comparing outcomes between programmes very difficult. Furthermore, potential moderators and mediators can be difficult to identify as programmes target different things and numbers in research studies are relatively small. This leaves the field relatively blind with regard to who responds best to DP treatment and who does not.

To better understand the differences in DP treatment models and how this may impact outcome this review aims to:

1. examine differences in DP treatment models
2. review available outcome data

From this review potential targets of future research and DP design can be targeted.

## Method

A systematic scoping review methodology (28) was used to explore the existing research into DPs for adolescents with eating disorders. This was identified as the most appropriate methodology given the heterogeneity of existing research and the broad aims of this review. Current scoping review guidelines (29) and the PRISMA guidance (30) were used to conduct this review.

### Search Strategy

Four databases (PsycInfo, Embase, Medline, CENTRAL) were searched using variations of the terms “eating disorder” and “day programme” and “adolescent” on 17th December 2020 (see Supplementary Material for exact search terms). Additional hand-searches of articles, reference lists and the internet were also performed.

### Selection Process

**Figure 1:**
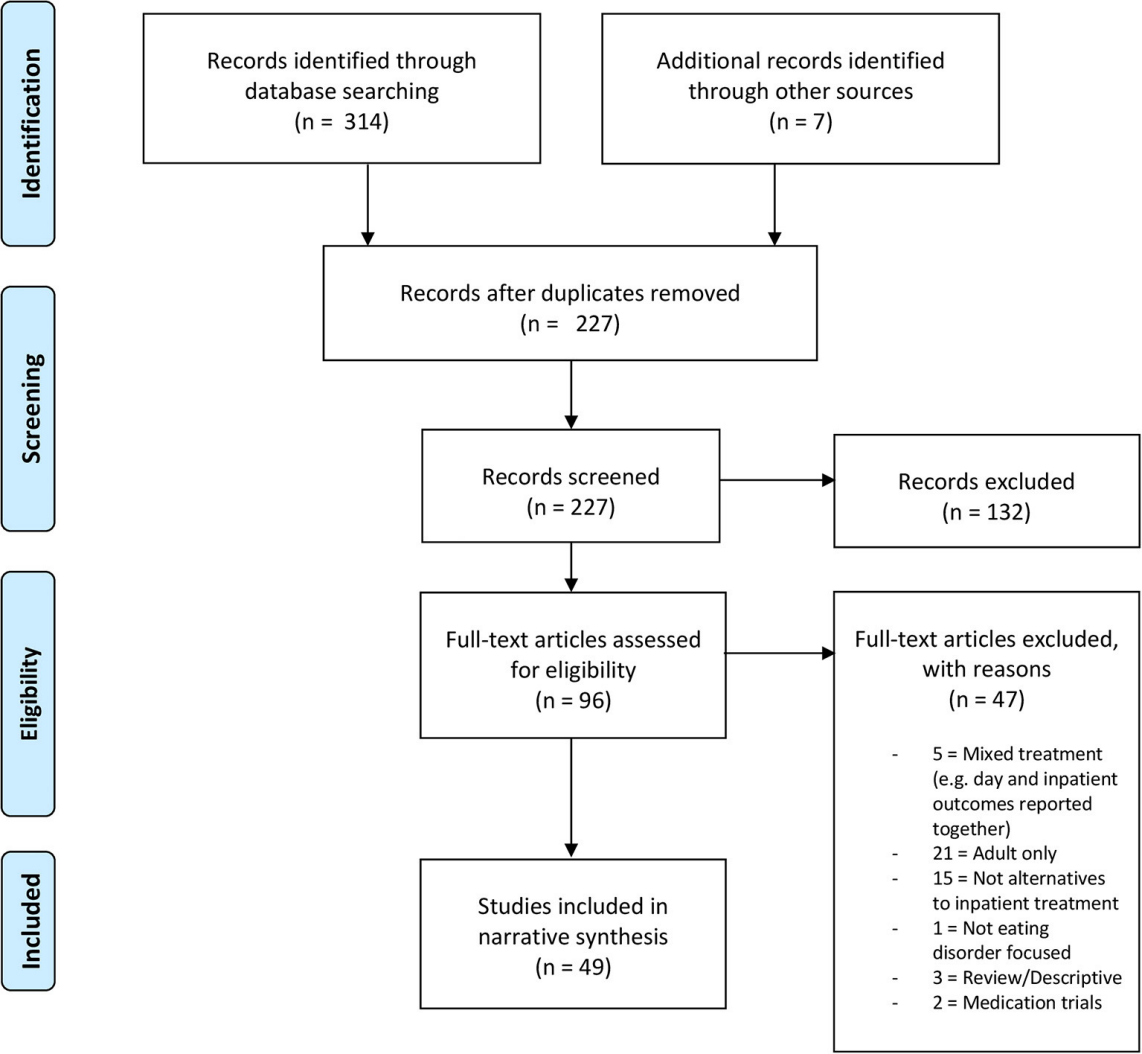
PRISMA flowchart.

Eligibility criteria for this review were determined a priori (see Table 1). After completing the initial search, duplicates were deleted and the remaining titles and abstracts reviewed by JB and MS. The remaining full-text citations were screened for eligibility by both authors before reaching consensus at the included papers in this synthesis (see Figure 1 for PRISMA flowchart). Zotero software was used in this process.

**Table 1:** Scoping review eligibility criteria.

	**Included**	**Excluded**
Publication type	-Peer-reviewed journal articles - Book chapters	-Conference abstracts -Unpublished dissertations
Language	-English	-non-English language
Study objectives	-Explicit focus on theoretical models and outcomes of adolescent day program treatment for eating disorders - Focus on programs that are alternatives to inpatient treatment	Explicit focus on day program treatment for adult day programs -Explicit focus on inpatient or outpatient treatment, -Explicit focus on intensive outpatient program only (<5 days per week, < half day per contact) -Integrated program where the day program component is not explicitly reported on - Medication focused
Methodology	- Quantitative	-Review articles
	-Qualitative	-Meta-analyses
	-Mixed methods	
Design	-Any	-None
Sample	-Child and adolescent	-Adult only
	-Mixed child, adolescent and adult	-Age 16 and over (without separate reporting on adolescent sample)

### Data Charting and Categorization

All included articles were charted according to two main categories: programme characteristics and study methodology characteristics. Program characteristics included location, treatment model, age range included, eating disorder diagnoses treated, and amount of contact per week. Study design characteristics included the year of publication, sample size, and study methodology. Each programme was then categorized as being family-focused or more individually focused. Programmes were categorized as family focused if they named a family therapy treatment model as primary, or if significant family involvement was required during treatment (see Supplementary Material for full coding criteria). The data-charting form was jointly developed by JB and MS to determine which variables to extract. This was then completed by both authors via an iterative process in repeated consultation. This information was used to inform the narrative synthesis of eligible studies.

## Results

### Study Selection and Characteristics

Three-hundred-and-fourteen papers were initially identified through the systematic literature search. Screening was performed according to the eligibility criteria outlined in Table 1. Forty-nine studies were determined to be eligible for this review (Figure 1). Full details and characteristics of all included studies are presented in Table 2.

**Table 2:** Study characteristics, programme details and outcomes.

**References**	**N (%F)**	**Study design**	**Mean age in years (SD, range)**	**Diagnosis**	**^∧^Admis. criteria/referral source**	**^∧^Aims/disch. criteria**	**Therapeutic model(s)**	**Treatment intensity**	**Mean length of stay (SD, range)**	**Baseline data mean (SD)**	**Discharge outcome data mean (SD)**	**Follow-up outcome data months (n, % baseline sample) & mean (SD)**
**California, USA**												
Brown et al. (31)	99 (97%)	Uncontrolled case series	15.8 (1.56, 11–19)	AN (100%)	W (<85% EBW)	WG (100% EBW), AWT, R	Family focused [FBT, DBT]	6–10 h/d 6 days/wk	92.9 days (NR, 29–281)	%EBW: 79.2 (NR) EDE-Q(G): 3.1 (NR)	%EBW: 94.2 (NR)^***^ EDE-Q(G): 1.8 (NR)^***^	6 months (*n* = 41, 41%) %EBW: 94.3 (NR) *ns*^§^ EDE-Q(G): 1.8 (NR) *ns*^§^*(FU weight self-report)*
Parks et al. (32)	29 (%NR)	Qualitative	16.6 (2.2, 12–21) *[all <18 during treatment]*	AN-R (34%) AN-BP (21%) BN (27.8%) OSFED (17.2%)	NR	NR	Family focused [FBT, DBT]	10 h/d 6 days/wk	NR	NR	NR	NR
Reilly et al. (33)	59 (49%)	Sample description	10 (NR, 6–12)	ARFID (100%)	MS, S	AWT, G	Family focused [FBT, DBT]	6 h/d 5 days/wk	NR	%IBW: 85.4 (7.0) [SE group] 86.8 (8.5) [FOC group] 82.8 (5.2) [LA group] ED symptoms NR	NR	NR
Reilly et al. (34)	265 (93%)	Uncontrolled case series	15.7 (1.71, 11–21)	AN-R (58%) AN-BP (13%) BN (14%) ARFID (6%) BED (1%) OSFED (7%)	MS, S, NG	AWT, I, R	Family focused [FBT, DBT]	6–10 h/d 6 days/wk	73.1 days (NR, NR)	%EBW: 87.5 (10.3) [AN group] EDE-Q(G): 3.2 (1.8)	%EBW: 99.7 (9.1)^***^ [AN group] EDE-Q(G): 2.0 (1.6)^***^	6 months (*n* = 93, 35%) %EBW: 98.2 (11.1) *ns*^§^ [AN group] EDE-Q(G): 2.0 (1.6) *ns*^§^ 12 months (*n* = 77, 29%) %EBW: 99.1 (10.1) *ns*^§^ [AN group] EDE-Q(G): 1.7 (1.6) *ns*^§^ *(FU weight self-report)*
**Georgia, USA**												
Freudenberg et al. (35)	151 (100%)	Uncontrolled case series)	22.5 (8.4, 13–57)	AN (49%) BN (51%)	NR	NR	Non-family focused [CBT, Psychod., DBT, MI, ACT, FT]	6 days/wk	13.7 weeks (9.5, 2–57) [AN group] 13.1 weeks (10.4, 1–45) [BN group]	99.4lbs (11.0, NR) [AN group] EDI-2: 7.6 (4.0) [AN group] 9.6 (6.8) [BN group]	108.9lbs. (13.1, NR)^**^ [AN group] EDI-2: 3.2 (2.9)^**^ [AN group] 3.9 (3.7)^**^ [BN group]	NR
Schaffner and Buchanan, (36)	77 (100%)	Uncontrolled case series	21.4 (6.7, 14–40)	*Eating disorders*	NR	NR	Non-family focused [CBT, Psychod., DBT, Art therapy, social skills, FBT for ado. AN]	3.5–7.5 h/d 6 days/wk	12.8 weeks (8.5, 1-43)	117.7lbs (33.3) EDI-2: 8.1 (3.8)	124.5lbs (30.1)^***^ EDI-2: 3.05 (2.8)^***^	NR
Schaffner and Buchanan, (36)	196 (98%)	Uncontrolled case series	22.6 (7.8, 13–51)	AN (%NR) BN (%NR) EDNOS (%NR) [purging: 81.5% bingeing: 77.4%]	NR	G	Non-family focused [CBT, BT, Art therapy, social skills]	3.5–7.5 h/d 6 days/wk	13.6 weeks (10.3, 1–60)	Weight NR EDI-2: 8.4 (3.9)	Weight NR EDI-2: 3.5 (3.2)^***^	NR
**Illinois, USA**												
Hayes et al. (37)	1,200 (93%)	Uncontrolled case series	21.2 (10.8, 11–68)	AN (19%) BN (12%) BED (12%) OSFED (56%)	S	NR	Non-family focused [CBT-E, DBT, ACT]	6 h/d 5 days/wk	19.2 days (12.4, NR)	zBMI: = −1.39 (0.95) [ <20 yrs, AN group] BMI: 17.5 (2.2) [>20 yrs, AN group] EDE-Q(G): 3.5 (1.5)	zBMI: −0.089 (0.84)^***^ [ <20 yrs, AN group] BMI: 18.6 (2.0)^***^ [>20 yrs, AN group] EDE-Q(G): 2.3 (1.5)^***^	NR
**Michigan, USA**												
Berona et al. (38)	102 (92%)	Uncontrolled case series	16.4 (2.9, 11–24)	AN (77%) “subthreshold AN” (23%)	NR	NR	Family focused [FBT]	6 h/d 5 days/wk	27.8 days (4.7, NR)	BMI: 16.3 (1.4) [rapid gain grp] 17.4 (2.1) [mod. gain grp] 18.1 (2.5) [slow gain grp] EDE-Q(G): 2.9 (1.6)	Lbs. gained +16.7 (3.4) [rapid gain grp] +8.6 (3.0) [mod. gain grp] +3.1 (2.2) [slow gain grp] EDE-Q(G): NR	No FU
Homan et al. (39)	113 (92%)	Uncontrolled case series	14.4 (1.7, NR) [ado.] 19.6 (1.57, NR) [YA]	AN (79%) EDNOS (21%)	MS	AWT	Family focused [FBT, CBT, DBT, CRT]	6 h/d 5 days/wk	21.8 days (12.9, NR)	BMI: 17.6 (2.2) EDEQ (global): 2.9 (1.8)	Means NR (Authors report both adolescents and young adults show improvements in symptoms during DP)	3 months (n, % NR) *DP led to symptom improvement that was maintained at follow up for adolescents, but not for young adults. Means NR*.
Hoste (40)	28 (89%)	Uncontrolled case series	16.6 (3.5, 8–24)	AN (71%) EDNOS-R (29%)	PP/SD	WG (90–95% EBW)	Family focused [FBT]	6 h/d 5 days/wk	31.7 days (13.9, 13–76)	%EBW: 82.1 (9.6) EDE-Q(G): 3.2 (1.9) %EBW: 81.6	%EBW: 93.1 (6.5) ^***^ [completers (*n* = 21)] EDE-Q(G): 1.9 (1.4)^**^	No FU
Rienecke (41)	53 (%NR)	Uncontrolled case series	“adolescents” (M, sd, range NR)	AN (67.9%) ARFID (13.2%) OSFED (18.9%)	PP/SD	AWT	Family focused [FBT]	6 h/d 5 days/wk	25 days (10.9, NR)	NR	NR *[no change in parental marital satisfaction during DP treatment]*	No FU
Rienecke (42)	87 (91%)	Uncontrolled comparison study (dropout [*n* = 19] vs. completers [*n* = 68])	14.1 (1.7, 10–18) [ado.] 19.6 (1.6, 19–24) [YA]	AN (100%)	PP/SD	AWT	Family focused [FBT]	6 h/d 5 days/wk	25.1 days (12.9, 1–74)	%EBW: 84.96 (7.7) [ado.] BMI: 17.54 (1.7) [YA] EDE-Q(G): NR	Completers: %EBW: 99.2 (10.7) [ado.] BMI: 19.8 (1.0) [YA] Dropouts: %EBW: 89.1 (10.3) [ado.] BMI: 18.9 (1.6) [YA] EDE-Q(G): NR	No FU
Rienecke (42)	3 (33%)	Case study	10.7 (3.1, 8–14)	ARFID (100%)	PP/SD	AWT	Family focused [FBT]	6 h/d 5 days/wk	20 days (1.7, 19–22)	BMI: 15.4 (1.3) ED measure NR	BMI: 16.7 (1.5) ED measure NR	No FU
Rienecke and Ebeling (43)	26 (89%)	Uncontrolled case series	15.5 (2.2, 12–19)	AN (46%) EDNOS-R (54%)	PP/SD	AWT	Family focused [FBT]	6 h/d 5 days/wk	27.6 days (10.9, NR)	%EBW: 88.1 (12.6) EDE-Q(G): 2.3 (1.6)	%EBW: 101.5 (14.8)^***^ EDE-Q(G): 2.0 (1.4) (ns)	No FU
Rienecke and Richmond (44)	26 (96%)	Uncontrolled case series	16.6 (3.2, 11–22)	AN (77%) EDNOS-R (23%)	PP/SD	WG (90–2% EBW), AWT	Family focused [FBT]	6 h/d 5 days/wk	28.2 days (14.6, NR)	%EBW: 80.9 (6.2) EDE-Q(G): 3.3 (1.7)	%EBW: 92.8 (5.1)^***^ EDE-Q(G): 1.8 (1.4)^**^	3 months (*n* = 25–26, 96–100%) %EBW: 97.7 (5.0)^**^^§^ [*n* = 25] EDE-Q(G): 1.5 (1.5) *ns^§^* [*n* = 26] *(FU weight self-report)*
Rienecke et al. (45)	56 (93%)	Uncontrolled case series	15.8 (2.9, 12–24)	AN (73%) EDNOS-R (27%)	PP/SD	WG (90% EBW), AWT	Family focused [FBT]	6 h/d 5 days/wk	27.6 days (12.1, NR)	%EBW: 82.6 (7.4) EDE-Q(G): 3.4 (1.7)	93.0% EBW (5.2)^***^ EDE-Q(G): 2.2 (1.4)^***^	No FU
Smith et al. (46)	51 (94%)	Uncontrolled case series	13.94 (NR, 9–17)	AN-R (70.6%) AN-BP (7.8%) A-AN (21.6%)	PP/SD	WG (90–95% EBW), AWT	Family focused [FBT, CBT, DBT, CRT]	6 h/d 5 days/wk	35.6 days (11.94, NR)	%EBW: 82 (6) EDE-Q(G): 2.4 (1.7)	%EBW: 93 (3)^**^ EDE-Q(G): 2.1 (1.5) (ns)	No FU
Van Huysse et al. (47)	70 (91%)	Uncontrolled case series	15.5 (2.6, 10–19)	AN (100%)	PP/SD	WG (>95%), R	Family focused [FBT]	6 h/d 5 days/wk	29.6 days (10.6, 10–74)	%mBMI: 80.0 (5.7) EDE-Q(G): 2.0 (1.5)	%mBMI: 91.9 (5.9)^**^ EDE-Q(G): 1.6 (1.3)*	No FU
**Missouri, USA**												
Fewell et al. (48)	423 (95%)	Uncontrolled case series	23.7 (9.5, 11–60)	AN (62.2%) BN (15.6%) BED (2.4%) ARFID (1.2%) OSFED (6.6%) EDNOS (12%)	NR	NR	Non-family focused [CBT, DBT, art therapy, music therapy, some FT]	NR	49.5 days (27.1, 7-120 days)	BMI: 17.7 (0.2) [AN group] EDE-Q(G): 4.0 (1.5)	BMI: 20.4 (0.2)^***^ [AN group] EDE-Q(G): 2.6 (1.5)^***^	12 months (*n* = 65, 15%) BMI: 20.61 (0.18) *ns*^§^ [AN group] EDE-Q(G): 2.9 (1.4)^*^^§^ *(sig. increase from discharge to FU)*
**New Jersey, USA**												
Huryk et al. (49)	326 (%NR)	Uncontrolled comparison study (FBT-DP [*n* = 138] vs. non-FBT-DP [*n* = 188])	15.7 (2.1) [FBT-DP] 15.9 (2.1) [DP] Range total sample 8–21	AN (74%) BN (6%) OS/UFED (20%) ARFID (0.6%)	NR	NR	Family focused [FBT, DBT, yoga, art, body image group]	40 h/wk	29.4 days (18.9) [FBT-DP] 33.0 days (14.6) [non-FBT-DP] total: 31.44 (16.39, NR)	%EBW: 82.9 (9.5) [FBT-DP] 87.0 (13.4) [non-FBT-DP] (84.6 (11.5) [total sample])	NR	No FU
**New York, USA**												
Dancyger et al. (50)	82 (100%)	Uncontrolled comparison study (orthodox [*n* = 8] vs. modern [*n* = 74] Jews)	16.0 (2.3) [orthodox group] 18.0 (2.5) [other] Range total sample 12–18	AN (63%) BN (20%) EDNOS (17%)	PP/SD	G, AWT	Non-family focused [Integrative MDT approach]	8 h/d 5 days/wk	15.3 weeks (NR) [orthodox group] 10.4 weeks (NR) [modern group]	%IBW: 94% (NR) [orthodox group] 92% (NR) [modern group] EDI-2(DT): 18.2 (5.2) [orthodox group] 14.3 (6.2) [modern group]	%IBW 102% (NR) [orthodox group] 95% (NR) [modern group] EDI-2(DT): NR	No FU
Dancyger et al. (50)	82 (98%)	Uncontrolled case series	17.9 (NR, 12–30)	AN (63.4%) BN (19.5%) EDNOS (17.1%)	PP/SD	AWT	Non-family focused [Integrative MDT approach]	8 h/d 5 days/wk	15 weeks (16.9, NR)	%IBW: 87 (NR) [AN group] 93 (NR) [EDNOS group] 112 (NR) [BN group] EDI-2(DT): 15.4 (5.7) [AN group] 16.8 (5.7) [EDNOS group] 10.5 (6.5) [BN group]	%IBW (sd NR): 91 [AN group] 98 [EDNOS group] 110 [BN group] EDI-2(DT): NR	No FU
deGraft-Johnson et al. (51)	198 (96%)	Uncontrolled case series	17.7 (NR, 12+)	AN (53%) BN (8%) EDNOS (39%)	PP/SD	AWT	Non-family focused [Integrative MDT approach]	8 h/d 5 days /wk	2.6 weeks (NR, 1–8)	17.8 BMI (NR)	Kg: +0.95 [all] Kg: +1.15 [AN group] (BMI, sd NR)	No FU
Wisotsky et al. (52)	65 (100%)	Uncontrolled case series	18 (3.3,12-27)	AN (65%) BN (18%) EDNOS (17%)	NR	NR	Non-family focused [Integrative MDT approach]	8 h/d 5 days/wk	Mean NR (range 4–394 days)	NR	NR	No FU
**Ohio, USA**												
Martin-Wagar et al. (53)	87 (92%)	Uncontrolled case series	14.9 (NR, 12–18)	AN-R (71%) AN-BP (29%)	PP	WG (>95%EBW), AWT	Family focused [FBT, CBT, DBT]	8 h/d 5 days/wk	7.4 weeks (4.7, 0–22.5)	%EBW: 82.83 (6.89) EDE-Q(G): 3.3 (1.8)	%EBW: 98.0 (9.3) EDE-Q(G): NR	No FU
**Pennsylvania, USA**												
Bustin et al. (54)	30 (87%)	Uncontrolled case series	12.8 (2.0, NR) “*adolescents”*	AN (33%) BN (7%) EDNOS (60%)	NR	NR	Family focused [As per Ornstein et al. (57)]	6-8 h/d 5 days/wk	33.3 days (9.9, NR)	%IBW: 86 (NR) ChEAT (total): 24.7 (NR)	%IBW: 96 (NR)^***^ ChEAT (total): 11.8 (NR)^***^	No FU
Bryson et al. (55)	62 (89%)	Uncontrolled comparison study (ARFID vs. AN in DP with FU)	11.4 (1.6) [ARFID group] 14.1 (1.5) [AN group] (range 7–17)	AN (68%) ARFID (32%)	S	AWT	Family focused [FBT, CBT, BT, ERP]	8.5 h/d 5 days/wk	Weeks: 6.8 (3.7) [ARFID group] 11.2 (5.3) [AN group]	%mBMI: 84.9 (7.9) [ARFID group] 81.6 (8.9) [AN group] ChEAT (total): 17.6 (14.2) [ARFID group] 9.2 (11.9) [AN group]	%mBMI:^***^ 94.0 (8.2) [ARFID group] 95.5 (6.9) [AN group] ChEAT (total):^***^ 33.6 (16.4) [ARFID group] 12.2 (11.5) [AN group]	30 months (*n* = 59, 95%) %mBMI: 95.1 (8.6) *ns^§^* [ARFID group] 97.9 (11.1) *ns^§^* [AN group] ChEAT (total): 5.8 (3.2)^*^^§^ [ARFID group] 9.0 (8.7)^*^^§^ [AN group]
Lane-Loney et al. (56)	81 (74%)	Uncontrolled case series	10.9 (2.2) [fear group] 13.1 (2.1) [appetite group] 11.5 (2.0) Co-primary group]	ARFID (100%)	MS, PP	AWT	Family focused [FBT, CBT]	5 days/wk	Days: 28.5 (11.0, NR) [FOC group] 22.9 (8.4, NR) [LA group] 29.9 (15.9, NR) [co-primary]	%mBMI: 88.3% (15.03) [FOC group] 85.6 [10.9] [LA group] 79.9 (89.6) [co-primary group] ChEAT(OC): 7.3 (3.7) [FOC group] 7.1 (5.5) [LA group] 7.35 [co-primary group]	%mBMI:^**^ 97.3 (14.3) [F0C group] 95.3 (10.1) [LA group] 79.9 (89.6) [co-primary group] ChEAT(OC)^**^: 3.7 (4.0) [FOC group] 5.5 (4.0) [LA group] 4.2 (3.3) [co-primary group]	No FU
Nicely et al. (57)	173 (92%)	Descriptive	13.5 (2.03, 7.2–16.9)	AN (53.8%) BN (11.6%) ARFID (22.5%) OS/UFED (12.1%)	NR	NR	Family focused	6-8 h/d 5 days/wk	NR	%mBMI 87.1 (13.0) [ARFID group] 82.6 (9.2) [AN group] 108.1 (19.5) [BN] 93.2 (6.8) [OS/UFED group] ChEAT (total): 14.9 (2.1) [ARFID group] 27.5 (17.3) [rest of group]	n/a	No FU
Ornstein et al. (58)	30 (87%)	Uncontrolled case series	12.8 (2, 8–16)	AN (33%) BN (7%) EDNOS (60%)	S, PP	AWT	Family focused	6-8 h/d 5 days/wk	33.3 days (13.4, NR)	%IBW: 86 (10) ChEAT (total): 20 (NR)	%IBW: 96% (7)^***^ ChEAT (total): 9.0 (NR)^***^	No FU
Ornstein et al. (59)	130 (92%)	Uncontrolled comparison study (ARFID vs other EDs in DP)	13.5 (2.1, 7–17)	AN (52.3%) BN (11.5%) ARFID (24.6%) OS/UFED (11.5%)	S, PP	AWT	Family focused [FBT, CBT, BT]	6-8 h/d 5 days/wk	Weeks in DP: 7.0 (3.4, NR) [ARFID group] 11.9 (4.2) [AN group] 8.9 (3.6) [BN group] 9.2 (3.7) [OS/UFED group]	%mBMI: 86.2 (10.0) [ARFID group] 82.9 (8.0) [AN group] 110.7 (21.1) [BN group] 93.4 (7.2) [OS/UFED group] ChEAT (total): 14.2 (12.8) [ARFID group] 30.5 (14.8) [AN group] 39.6 (19.1) [BN group]	%mBMI:^***^ 95.5 (8.0) [ARFID group] 95.2 (5.5) [AN group] 109.2 (17.4) [BN group] 98.4 (5.2) [OS/UFED group] ChEAT (total):^***^ 9.8 (10.5) [ARFID group] 11.6 (10.5) [AN group] 13.9 (13.0) [BN group] 14.0 (12.0) [OS/UFED group]	No FU
										25.0 (18.6) [OS/UFED group]		
Zickgraf et al. (60)	83 (76%)	Descriptive	11.38 (NR, 8–17)	ARFID (100%)	S	NR	Family focused [FBT, CBT, BT, ERP]	8.5 h/d 5 days/wk	NR	%MBW 95.2 (28.7) [SE group] 83.5 (11.4) [LA group] (16.1) [FOC group] 80.1 (9.6) [co-primary] Selective: 95.23%mBMI (28.71) ED sympt. NR	n/a	No FU
**Wisconsin, USA**												
Bean et al. (61)	16 (88%)	Uncontrolled comparison study (FBT-DP [*n* = 9] vs. non-FBT-DP [*n* = 7])	15.4 (2.6, 12–20)	AN-R (100%)	NR	AWT	Family focused [FBT, CBT, IPT]	2–6 h/d 5 days/wk	Weeks: 11.6 (5.6, 5–24) [FBT-DP] 11 weeks (5.2, 4–18) [non-FBT-DP]	BMI: 16.9 (NR) [FBT-DP] 16.2 (NR) [non-FBT-DP] EDE-Q(G): 3.8 (NR) [FBT-DP] 2.6 (NR) [non-FBT-DP]	BMI:^*^ 19.6 (NR) [FBT-DP] 19.2 (NR) [non-FBT-DP] EDE-Q(G): 1.6 (NR)^*^ [FBT-DP] 1.3 (NR) (ns) [non-FBT-DP]	No FU
**Canada**												
Girz et al. (62)	17 (100%)	Uncontrolled case series	16.1 (1.0, 13–18)	AN-R (24%) BN (35%) EDNOS-R (35%) EDNOS-BP (6%)	PP/SD, MS	AWT	Family focused [FBT]	5 days/wk	149.76 days (30.34, NR)	%IBW: 88.0% (NR) EDI-3(DT): 49.2 (12.6)	%IBW: 16/17 reached 100% EDI-3(DT): 31.1 (13.1)^*^	No FU
Grewal et al. (63)	65 (94%)	Uncontrolled case series (completers [*n* = 38] vs. non-completers [*n* = 27])	15.6 (1.4, 13–18)	AN-R (60%) AN-BP (14%) BN (11%) BED (3%) EDNOS (12%)	W (> 80% GW), SD	WG (100% GW)	Family focused [FBT]	5 days/wk	200.4 Days (109.8, 42–517)	%GW: 91.7 (6.1) ED sympt. measure NR	%GW: 101.8 (7.7)^*^ [restrictive group only] ED sympt. measure NR	No FU
Henderson et al. (64)	65 (100%)	Uncontrolled case series	15.0 (1.3, 11–17)	AN (64%) BN (10%) EDNOS (26%)	SD	WG (>19 BMI), AWT	Family focused [FBT]	10 h/d 5 days /wk	14.8 weeks (6.0, NR)	BMI: 18.7 (2.4) EDI-2(DT): 16.1 (6.0)	BMI 20.5 (2.0)^***^ EDI-2(DT): 11.6 (7.4)^**^	6 months (*n* = 43–61, 66–95%) BMI 19.8 (2.2) ^***^^∧^ [*n* = 61] EDI-2(DT): 11.72 (7.3)^***^^∧^ [*n* = 43]
Ngo and Isserlin (65)	49 (100%)	Uncontrolled comparison study (completers [*n* = 14] vs. failures [*n* = 35])	15.3 (1.2, 13–17)	AN-R (69.4%) AN-BP (30.6%)	NR	WG (>92.5% IBW)	Non-family focused [CBT, art therapy, may have FT]	8 h/d 4 days/wk	81.9 days (61.7, NR)	%IBW: 84.1 (4.5) ED sympt. Measure NR	%IBW: 89.9 (5.4) ED sympt. Measure NR	No FU
Pennell et al. (66)	24 (96%)	Uncontrolled case series	15.4 (1.3, 13–17)	AN-R (42%) AN-BP (25%) BN (4%) EDNOS (21%) ARFID (8%)	PP/SD, S	G, AWT	Family focused [FBT, DBT]	6–10 h/d 5 days/wk	8.8 weeks (6.6, 2–35)	%IBW: 94.8 (8.2) ED sympt. Measure NR	IBW: 99.5 (8.0)88 5/7 abstinent from binge-purge behaviors	No FU
**Spain**												
Lazaro et al. (67)	160 (94%)	Uncontrolled comparison study (AN-r [*n* = 116] vs. BN-r [*n* = 44])	15.5 (1.2, 13–18)	AN (59%) BN (18%) EDNOS (23%)	PP/SD, S	AWT	Non-family focused [CBT, BT]	6.5 h/d 5 days/wk	3 months (NR)	BMI: 18.3 (1.2) [AN-rd group] 20.3 (3.3) [BN-rd group] EAT-40: 49.9 (26.0) [AN-rd group] 50.9 (18.1) [BN-rd group]	BMI: 19.2 (NR) [AN-rd group] 20.6 (NR) [BN-rd group] EAT-40 NR	No FU
Serrano-Troncoso et al. (68)	77 (94%)	Uncontrolled case series	14.4 (1.6, 11–17)	AN-R (94%) AN-BP (6%)	MS, NG (NICE, 2017)	WG (>90% EBW), AWT	Non-family focused [CBT, BT, parenting elements]	11 h/d 5 day/wks	28.9 days (18.5, NR)	BMI: 17.2 (NR) ED symptom measure NR	BMI: 17.9 (NR)^***^ ED symptom measure NR	12 months (*n* = 70, 91%) BMI: 19.3 (NR)^***^^∧^
**UK**												
Baudinet et al. (69)	130 (95%)	Uncontrolled case series	15.0 (1.5, 11–18)	AN-R (84%) AN-BP (5%) A-AN (5%) OSFED (6%)	PP/SD, S, MS	AWT	Family focused [FT-AN, RO-DBT, CBT, CRT]	6 h/d 5 days/wk	13.4 weeks (5.9, 1–30)	%mBMI: 82.4 (8.5) EDI-3(DT): 18.3 (8.5)	%mBMI: 89.5 (8.6)^***^ EDI-3(DT): 15.2 (8.9)^*^	No FU
Pretorius et al. (70)	24 (96%)	Uncontrolled case series	15.6 (1.4, 12–17)	AN (71%) EDNOS (29%)	PP/SD, S, MS	AWT	Family focused [FT-AN, DBT, CBT, CRT]	6–8 h/d 5 days/wk	NR	%mBMI: 78.5% (9.9) ED symptom measure NR	%mBMI: 82.6 (9.4)	No FU
Simic et al. (71)	105 (95%)	Uncontrolled case series	15.5 (1.5, 11–18)	AN-R (91%) AN-BP (1%) ARFID (5%) OSFED (3%)	PP/SD, S, MS	AWT	Family focused FT-AN, DBT, CBT, CRT]	6 h/d 5 days/wk	28.4 days (13.6, NR)	%mBMI: 79.9 (8.69) EDE-Q(G): 3.6 (1.4)	%mBMI: 85.0 (9.10)^***^ EDE-Q(G): 2.6 (1.5)^***^	6 months (*n* = 86, 82%) %mBMI: 88 (10.6)^**^^§^ EDE-Q(G): NR
**Germany**												
Herpertz-Dahlman et al. (26)	172 (100%)	RCT (DP [*n* = 87] vs. IP [*n* = 85] after 3 weeks of IP)	15.2 (1.5, 11–18)	AN-R (82%) AN-BP (18%)	S, PP, MS	AWT	Non-family focused [CBT, BT, some FT]	8.5 h/d 5 days/wk	Weeks: 16.5 (7.0) [DP] 14.6 (6.0) [IP]	%EBW: 74.4 (7.0) [DP group] 75.4 (6.2) [IP group] EDI-2 (global): 248.8 (58.2) [DP group] 272.5 (59.4) [IP group]	%EBW:^***^ 89.0 (3.8) [DP group] 88.1 (4.7) [IP group] EDI-2 (global): NR	12 months (post-randomization) (*n* = 142–161, 83–94%) %EBW:^***^^∧^ [*n* = 161] 89.0 (3.8) [DP group] 88.1 (4.7) [IP group] EDI-2 (global): [*n* = 143] 248.2 (71.1) [DP group] 256.2 (78.2) [IP group]
**Australia**												
Goldstein et al. (72)	28 (100%)	Uncontrolled case series	15 (12–18)	AN (79%) EDNOS (21%)	MS, SD	Fixed length	Non-family focused [CBT, narrative therapy, distress tolerance]	3.5 days/wk (18 h/wk)	10 weeks fixed length	%IBW: 81.6 (7.7) EDI-3(DT): 13.8 (9.1)	%IBW: 84.2 (10.0)^**^ EDI-3(DT): 10.1 (8.3)^**^	6 months (*n* = 17–20, 61–71%) %IBW: 88.6 (12.1)^**^^∧^ [*n* = 20] EDI-3(DT): 5.88 (6.85)^**^^∧^ [*n* = 17]
Green et al. (73)	42 (100%)	Uncontrolled case series	16.7 (2.9, 12–24)	AN-R (83%) AN-BP (17%)	MS	AWT	Non-family focused [CBT]	5.75 h/d 5 days/wk	22 weeks (NR, 0–52)	BMI: 17.0 (1.5) EDI-3(DT): 57.1 (28.8)	BMI: 18.9 (1.7)^**^ EDI-3(DT): 31.0 (26.0)^***^	No FU
**Israel**												
Danziger et al. (74)	32 (97%)	Uncontrolled case series	14.5 (2.0, 10–17.5)	AN (100%)	S	WG (within 1 kg of IBW)	Family focused MDT approach	14 h/day	NR	38 kg (6.0) ED symptom measure NR	47.25 kg (6.2) body image disturbance disappeared for 19/45	9 months (*n* = 32, 100%) 27/31 retained IBW
Danziger et al. (75)	45 (93%)	Uncontrolled comparison (psychotherapy [*n* = 21] vs. not [*n* = 24] in first 2 months of DP)	14.7 (2.0,10-17.5)	AN (100%)	S	AWT	Family focused MDT approach	14 h/day	NR	37.4 kg (6.8) [therapy group] 39.1 kg (5.3) [no therapy group] ED symptom measure NR	42.8 kg (7.8) [therapy group] 46.4 kg (5.8) [no therapy group] (no therapy sig > therapy group^**^)	13.5 months (n, % NR) +10.4 kg (4.3) [therapy group] + 11.0 kg (5.50) [no therapy group]

* *p < 0.05*;

** *p < 0.01*;

*** *p < 0.001*.

∧ *Codes for admission and discharge criteria: AWT, agreement with team; G, reaching goals; I, insurance constraints; MS, medically stable; NG, as per a national guideline; PP, poor progress; R, remission; S, severity/acuity; SD, step-down from inpatient care; W, weight cut/off; WG, weight goal*.

*+Significance testing for baseline to follow up difference*.

§ *Significance testing for discharge to follow up difference*.

NR, not reported.

*CT, acceptance and commitment therapy; ado., adolescent; AN, anorexia nervosa; AN-rd, anorexia nervosa and related disorders; ARFID, avoidant/restrictive food intake disorder; Ax, assessment; BED, binge eating disorder; BMI, body mass index; BN, bulimia nervosa; BN-rd, bulimia nervosa and related disorders; BT, behavior therapy; CBT, cognitive behavioral therapy; ChEAT, Children's Eating Attitudes Test; ChEAT(OC), oral control subscale of ChEAT; CRT, cognitive remediation therapy; DBT, dialectical behavior therapy; DP, day program; Dx, diagnosis; EAT-40, Eating Attitudes Test; ED, eating disorder; ED-Rs, restrictive eating disorders; EDE-Q, Eating Disorder Examination Questionnaire; EDI, Eating Disorder Inventory; EDNOS, eating disorder not otherwise specified; EDNOS-R, eating disorder not otherwise specified characterized by restriction; EOT, end of treatment; FBT, family based treatment; FOC, feat of aversive consequences; FT-AN, family therapy for anorexia nervosa; FU, follow up; IBW, ideal body weight; IOP, intensive outpatient program; IP, inpatient; LA, limited appetite or lack of interest in eating; MDT, multi-disciplinary team; MI, motivational interviewing; OSFED, other specified feeding and eating disorder; OSFED-R, other specified feeding and eating disorder characterized by restriction; PHP, partial-hospitalization program; PMM, predictors, moderators or mediators; Psychod., psychodynamic psychotherapy; RO-DBT, radically open dialectical behavior therapy; SE, selective eating due to sensory properties; UFED, unspecified feeding and eating disorder; UK, United Kingdom; YA, young adult*.

The field of adolescent DPs has changed significantly in the last 3–5 years. The vast majority (*n* = 43, 88%) of papers were published in the last decade and nearly half (*n* = 22, 45%) within the last 2 years. The majority of included studies were from the USA (*n* = 34, 69%) and used uncontrolled case series or retrospective chart review designs (*n* = 47, 96%). One qualitative study and one randomized controlled trial (RCTs) was identified. The latter compared DP treatment to inpatient treatment (26).

Sample sizes of the included studies varied considerably. Nearly a quarter (*n* = 12, 24%) had a very small sample size of 30 participants or less. Fifteen (31%) had a sample size larger than 100.

Approximately two-thirds of published papers reported on programmes whose treatment model was family focused (*n* = 34, 69%). Several treatment centers published multiple papers on different aspects of the same DP. Eighteen (37%) included studies appeared to be produced by two centers; one in Michigan, the other in Pennsylvania.

Across all 49 studies, the combined total sample reported on was 5,594 (mean age = 17.7 years, 93% female). Anorexia nervosa was the most common diagnostic group (*n* = 3,056, 57%), followed by unspecified eating disorders (*n* = 1,243, 23%). Importantly, this number is likely inflated as several studies reported on different aspects of the same programme and potentially used the same, or similar samples across different published studies. Programme and study characteristics are presented in Table 2 below.

Twenty-six (53%) of the studies reviewed included the assessment of symptoms of anxiety and/or depression. Twenty-one (43%) reported rates of comorbid diagnoses, which ranged from 14% (72) to as high as 70–80% (37, 60). Only two studies reported comorbidity rates <30% (68, 72).

### Narrative Synthesis

#### Adolescent Eating Disorder Day Program Design

One of the key differences between the DPs reviewed was their design and theoretical framework. Most DPs offer a combination of individual, family, multi-family and group-based interventions, which are often combined into a structured daily timetable. Clear rules and expectations regarding participation, symptom management and weight gain/maintenance depending on individual presentation are also typically established before treatment commences. All programmes offer meal support several times per day, which is a core component of any DP. However, beyond this structure large variability existed in terms of the age range, diagnoses treated and treatment models informing practice. See Table 2 for details.

#### Population: Age, Presentation, and Diagnosis

Full details of each programme are presented in Table 2. The majority of studies (*n* = 31, 63.3%) exclusively report on children and adolescents up to 19 years of age. Ten (20.4%) focus on adolescents and young adults together. The remainder (*n* = 8, 16.3%) mix all ages across the lifespan. Rarely did a programme admit primary school-age children, although children as young as six (33), seven (59), and eight (49) have been included in some studies.

Similarly, there is variability in the diagnostic mix of young people who attend eating disorder DPs. Many programmes provide treatment to young people with any eating disorder diagnosis (*n* = 21, 43%), although the literature indicates that even in mixed diagnostic samples the majority who attend DPs are diagnosed with anorexia nervosa or eating disorders that are primarily characterized by restriction and weight loss (see Table 2). Four (8%) describe or report outcomes for young people with Avoidant/Restrictive Food Intake Disorder (ARFID) exclusively.

#### Day Programme Admission and Discharge Criteria

There was large variability in the admission and discharge criteria described for DP treatment in the studies reviewed. The majority reported admitting people due to a lack of progress in outpatient treatment and/or high clinical acuity (*n* = 31, 63%). Medical stability was explicitly stated as an admission criterion by eleven (23%) studies. Two (4%) studies mention a specific weight criterion for entry into their study. Of these one required a minimum weight of 80% of the individual's goal weight or higher (63), while the other included adolescents with an estimated body weight of 85% or less (31). Only two (4%) studies refer directly to national guidelines when describing admission criteria and a quarter (*n* = 12, 24%) did not report on admission criteria.

Regarding reported DP treatment aims and discharge criteria, large variability between studies also exists. Twelve studies (24%) report a specific weight target or weight range (from 90 to 100% of the individualized target weight) for participants to reach before discharge. More commonly, discharge occurs following some clinical improvement and/or progress toward established goals, with readiness for outpatient treatment decided with the clinical team. One study (2%) described insurance constraints regarding treatment length. Eleven (22%) did not specify discharge criteria (see Table 2 for details). Some programmes also offer an additional tier of intervention between DP and outpatient care. In the USA particularly it has been common to offer both a partial hospitalization program (more intensive) and an intensive outpatient program (less intensive), within the same treatment center [e.g., (34, 37, 39, 40, 53)], which may be partially influenced by insurance requirements.

#### Day Programme Intensity and Length

Most studies describe operating 5 days per week (*n* = 37, 76%). Only a small number offer fewer (*n* = 2, 4%) or more days (*n* = 6, 12%), and four (8%) studies did not specify the number of days. Similarly, the number of treatment hours per day is typically six to eight (*n* = 30, 61%), but some programmes offer up to 11 (68) or 14 h per day (74, 75).

Treatment length is difficult to compare across all studies due to reporting differences. Twenty-five (51%) studies report length of stay in number days, whereas 15 (31%) reported it in weeks, one (2%) in months, and eight (16%) did not report a mean length of stay. Of those programs that reported length of stay in days, the majority (*n* = 15, 60%) reported a mean length between 25 and 40 days. For those that reported weeks or months, most (*n* = 12, 75%) reported a mean length of stay between 10 and 16 weeks, or ~3 months. In summary, the length of stay ranges from a month or less (37, 51, 76) to 6 months or more (62, 63). Only one study reported a fixed length of stay [10 weeks; (72)], and one reported a minimum stay [2 months; (67)]. See Table 2 for details.

#### Treatment Models and Responsibility for Change

Several DPs describe themselves as being either exclusively or predominantly based in one particular treatment model, such as family based treatment (FBT) or cognitive behavior therapy (CBT). Alternatively, some programmes mix two or more treatment models offering more integrative treatment. Others do not report on the specific treatment model(s) used, rather only report on the format of the treatment delivered (e.g., individual, group, family, multi-family, etc.). See Table 2 for details. Regardless of the model described, due to the large amount of contact time in DP treatment, it is rare for a programme to exclusively operate according to only one treatment model.

Perhaps the most important thing to consider regarding treatment model is the conceptualization of whom primarily holds the responsibility for change, the young person or the family. Given the adolescent developmental stage, almost all adolescent DPs include some family or parental involvement, however, their role varies. It ranges from being placed completely in charge of early change in treatment to taking a much more peripheral and supportive role throughout. A recent conceptual comparison of two specific adolescent eating disorder treatments, family based treatment [FBT; (77)] and enhance cognitive behavior therapy [CBT-E; (78)], highlighted fundamental differences in the two treatment approaches. Lock and Le Grange (77) state that family and parental involvement in the adolescent's therapy is necessary for treatment success. The family is viewed as a great resource in the treatment of their child. Conversely, the CBT-E model posits the illness belongs to the individual, who holds the responsibility for change (2).

These theoretical differences filter down into the way specific treatment models have been adapted for DP treatment. Hoste's (40) description of a FBT-informed DP in the USA heavily emphasizes the importance of the parents by placing them in charge of meals, encouraging parental persistence in the face of their child refusing food, not offering meal replacements and the less-directive role staff play to ensure they do not disempower parents. Alternatively, CBT-E based programmes prioritize involving the young people in decision making throughout the process, emphasizing the voluntary nature of the programme, with the view that this empowers the young person to take control over the process (18).

Not all adolescent eating disorder treatments or DPs hold such dichotomous views about the process of recovery. Engaging the adolescent and family are both formulated as essential to the recovery process in the broader form of family therapy for anorexia nervosa [FT-AN; (79)]. Programs that integrate family models with other treatment models offer intervention targeted at both individual and family factors, implying that both have some responsibility for change.

### Day Program Outcomes

#### Physical Health

It is now well-established that DP treatment is associated with improvements in weight for underweight adolescents. Every study reviewed that investigated weight gain reported a mean increase from assessment to discharge (see Table 2). This appears to be consistent internationally across programmes regardless of the treatment model or eating disorder diagnosis. Bryson et al. (55) found no difference in weight gain for adolescents with ARFID compared to anorexia nervosa. For adolescents attending all age DPs both groups appear to respond similarly. In two studies that included mixed adolescent and adult samples, no differences were found in the amount of weight gain for adolescents and adults by the end of treatment (35, 50). Nevertheless, no comparison of treatment response between adolescent and all-age programs has been made to date.

#### Eating Disorder and Comorbid Psychopathology

It is also widely reported that following DP treatment young people report reductions in a range of core eating disorder symptoms and cognitions, such as drive for thinness, shape and weight concerns and body dissatisfaction (see Table 2). Similarly, for those with binge/purge behaviors at assessment, reductions are reported by end of treatment (34–36, 58).

From the available data, it has been consistently reported that DP treatment is associated with improvements in symptoms of depression (35–37, 48, 56, 58, 62, 64, 69, 73), as well as anxiety and worry (36, 48, 58, 59, 62, 71, 73). There are some individual differences between studies in the pattern of improvements. For example, Henderson et al. (64) found that anxiety did not significantly improve during DP treatment itself but did significantly improve during the 6-month follow-up period. These findings are encouraging as comorbidity is high across the studies reviewed (typically ~30–70%) and the data suggests broader, more holistic recovery may be supported in DP treatment.

#### Psycho-Social Functioning

Some studies have also investigated broader change beyond psychopathology. Several programmes investigated more general psychosocial functioning, such as global functioning, social and school functioning, psychosexual adjustment, etc. Regardless of the aspect of functioning investigated or instrument used, all reported improvements during DP treatment (26, 37, 48, 73).

Similarly, adolescents report improvements in emotion regulation, emotional expression, cognitive flexibility, attachment relationships and social functioning at end of treatment (69, 71), although cognitive flexibility did not improve after a brief 4-week cognitive remediation group offered within the DP context (70). Significant improvements in self-esteem have been reported (67, 71). Lázaro et al. (67) specifically investigated change in self-esteem, social functioning and social skills during their DP treatment that included groups specifically targeting these domains. They found that adolescents generally improved in these domains over the course of their DP (mean duration = 3 months). However, there were differences in responding depending on diagnostic grouping. Adolescents with bulimia related disorders reported lower self-esteem and social skills at assessment but improved more during treatment compared to those with anorexia nervosa and related difficulties. All these factors are hypothesized to be core difficulties for people with eating disorders and may contribute to the maintenance of symptoms and impaired functioning.

#### Quality of Life and Motivation

Evidence is now suggesting that DP treatment is associated with improved quality of life and motivation to recover. After both brief and longer DP treatment adolescents report significant improvements in quality of life (37, 71). Furthermore, motivation and readiness to change improve across DP treatment, regardless of the treatment model (54, 72, 73). Higher motivation at assessment also predicted the amount of weight gain in one small (*N* = 42) Australian study (73).

#### Family Factors and Outcome

Compared to individual adolescent factors, relatively little attention has been given to parent, caregiver and family factors. Fourteen studies (29%) measured parental factors and no study included siblings or wider family members. The only qualitative study in the review reported that adolescents and families are initially unsure about family involvement in DP treatment, but this improves during treatment and most say that it is an important part of treatment upon reflection (32).

Family functioning was reportedly very poor at entry into one DP (52). Poorer functioning was also associated with increased eating disorder psychopathology (52). However, parental marital satisfaction, another marker of family functioning, was not associated with baseline illness severity or treatment dropout in another study (41).

Parental self-efficacy and readiness for change have also been investigated. Parental self-efficacy improved and caregiver burden reduced during treatment in one study (62). The authors noted that the timing of changes in perceived burden coincided with physical and psychological improvements for the adolescent (62). With regard to readiness for change, one study found that parents and adolescents report similar levels initially, but by the end of treatment adolescents are more ready for change than their parents (54).

Lastly, parental expressed emotion has also been investigated, although the data are mixed. Maternal expressed emotion reduced between baseline and discharge in one study (44), while paternal expressed emotion was reported to either stay the same (44) or reduce (39) across treatment. Whether this interacts with outcomes is not reported, although higher expressed emotion has been associated with a slower weight gain trajectory (38). Expressed emotion may also impact upon therapeutic alliance. In one study, higher maternal hostility toward their child was associated with poorer therapeutic alliance with the team/clinician, although this did not impact outcomes (45).

### Outcomes at Follow-Up

Increasingly, follow-up data are now being published (see Table 2 for details). Thirteen (27%) studies included follow-up data at different time intervals, including 3 months (39, 44), 6 months (31, 33, 34, 64, 71, 72), 9 months (75), 1 year (26, 34, 48, 55) and beyond (55, 75).

#### Three-Month Follow-Up

Treatment improvements are reported to be maintained between discharge and 3-month follow-up (39, 44). This includes maintenance of weight, eating disorder symptomatology and mood. In one study adolescent shape concerns continued to improve over this period (44). For parents, self-efficacy improvements were maintained or improved upon and emotional over-involvement reduced (44).

#### Six- and Nine-Month Follow-Up

At 6-month follow up outcome reporting is more varied. Two studies found that adolescents continue to gain weight during the 6- (71, 72) and 9-month follow-up periods (74). However, two other studies report a reduction in remission rates between discharge and 6-month follow-up (31, 34).

#### 12-Month Follow-Up and Beyond

At 12-month follow-up many adolescents continued to do well-physically and psychologically. Regarding weight, four studies reported that adolescents maintained their weight at 12 months or more post-discharge (34, 48, 55, 75) and one reported that weight continued to increase (68). DP treatment has also been shown to be equivalent to inpatient treatment for weight gain at 12 months from the start of treatment (26). By the 2.5 year mark from the start of treatment those who received DP treatment had higher BMI and significantly fewer relapses and admissions to hospital than those who received inpatient treatment (22), although the magnitude of these difference have not been specifically reported. Bryson et al. (55) also found that adolescents with restrictive eating disorders (anorexia nervosa and ARFID) maintain their weight at longer-term follow-up (mean length to follow up 30 months).

The pattern of change in eating disorder symptomatology beyond weight is more varied. Two family-focused, adolescent-only DPs reported that improvements were either maintained (34) or significantly improved upon at 12-month or more follow-up (55). Conversely, Fewell et al. (48) found a significant worsening of eating disorder symptoms, despite weight maintenance, at 12-months post-treatment in their all-age DP. Regarding comorbidity, Reilley et al. (34) reported that symptoms of depression and anxiety continued to improve at 6- and 12-month follow-up. Lastly, maternal and paternal expressed emotion (both criticism and emotional over-involvement) significantly reduce between admission and 12-month follow-up (80).

It is important to note substantial amounts of missing data in some studies at longer follow-up time points. One study reported 63.4% and 70.9% missing data at 6- and 12-month follow-up, respectively (34), while another reported 85% missing at 12 months (48). Furthermore, Bryson et al. (55) report that of those eligible for their follow-up study, only 45.3% consented to participate, highlighting the difficulty of obtaining complete follow-up data.

#### Treatment Drop-Out and Non-completion

Treatment completion, drop-out and treatment non-completion are defined very differently depending on the service. Some studies report on the rates of “non-completers,” which usually means there is disagreement between the individual, family and clinical team about readiness for discharge. This term, or “treatment failure,” is also used in some studies to refer to adolescents who do not meet a specified weight target by the end of treatment [e.g., (65)]. Others report on the number of people who are referred to inpatient treatment or higher levels of care as markers of poor outcome.

From the data available, most adolescents who start DP treatment will go on to complete it. Non-completion rates range from 8.9 (45) to 41.5% (63), although are most commonly reported at ~20% (26, 42, 58, 59).

#### Referral to Higher Levels of Care and Readmission Rates

When reported, rates of admission to inpatient from DP treatment range from ~5–35% (42, 49, 50, 54, 55, 59, 65, 68, 69, 71, 72) and readmission rates to DP range from ~3–20% (49, 66, 68, 69, 71). Huryk et al. (49) observed that the readmission rate to their DP significantly reduced from 12 to 3% after the integration of FBT principles into their DP.

### Predictors, Moderators, and Mediators of Day Program Outcomes

#### Age and Outcome

Age did not impact upon treatment outcome or need for higher levels of care in two adolescent family-focused DPs (39, 58). However, the picture is more mixed in all age programmes. Hayes et al. (37) found that younger participants had poorer outcomes in their large study (*N* = 1,200). In a smaller study (*N* = 82), however, this was not replicated (50).

#### Diagnosis and Outcome

Most studies do not have adequate numbers to explore differences in outcomes between different diagnostic groups. All adolescent, regardless of diagnosis, have been shown to benefit from treatment (35). However, participants diagnosed with anorexia nervosa (as opposed to bulimia nervosa or eating disorder not otherwise specified [EDNOS]) had worse outcomes in one very large (*N* = 1,200) study (37). In a much smaller study (*N* = 82) there were no differences in outcome according to diagnosis (50).

Within the cluster of restrictive eating disorder diagnoses (anorexia nervosa, ARFID, EDNOS-restrictive) the only available data are from family-focused DPs. Adolescents with ARFID have similar improvements in physical and psychological outcomes to those with anorexia nervosa (55, 59). This has not been investigated in all age programmes.

#### Eating Disorder Severity and Outcome

Several studies have investigated whether certain markers of eating disorder illness severity are associated with outcomes at the end of treatment. Some found that eating disorder symptom severity measured using self-report questionnaires (and other markers of illness severity, such as length of illness at assessment, presence of binge/purge behaviors, amount of weight loss at assessment) is associated with poorer outcomes at discharge from FBT-informed programmes (31, 53, 58, 63) and an all-age programme (48). Conversely, Ornstein et al. (58) found that eating disorder severity did not predict physical or psychological improvements in their family-focused DP. Additionally, Ngo and Isserlin (65) found that low body weight at admission (<85% ideal body weight) was not associated with poorer outcomes. Lastly, Homan et al. (39) found that most factors they investigated did not impact upon change in eating disorder psychopathology by end of treatment, including previous hospitalization or previous treatment.

One small study also demonstrated that adolescents who have very low desired ideal body weight targets (one marker of greater cognitive distortion) reported higher levels of restriction at the end of FBT-informed DP treatment (43). Furthermore, cognitive improvements in eating disorder symptoms were associated with reduced mealtime anxiety over the course of treatment in another study (46).

#### Comorbidity at Assessment and Outcome

Again, the data here are mixed. Two studies explored whether comorbidity at assessment impacts treatment outcome in FBT-informed programmes. Ornstein et al. (58) found that the severity of mood and anxiety symptoms at assessment was not associated with psychological or physical improvements at discharge. Homan et al. (39), however, noticed different patterns of responding depending on diagnosis. Adolescents with anorexia nervosa, compared to those with EDNOS, demonstrated greater treatment gains regardless of the level of depression at assessment. Adolescents with EDNOS showed treatment gains only at moderate or high levels of depression.

Within all-age programmes, inconsistent findings are also reported. Fewell et al. (48) found that severity of comorbid depression and worry symptoms were associated with worse outcomes in their programme. Conversely, Hayes et al. (37) found that those who were more depressed did better in their programme. It is important to note the large difference in programme length between these two programmes (49.5 vs. 19.2 days, respectively), as this may limit the comparability of these findings.

#### Family Factors and Outcome

Very little has been investigated regarding family factors and how these potentially impact upon DP treatment outcome. Ornstein et al. (58) found that neither intact families nor parental level of education predicted outcome in their programme. It has also been reported that parental engagement (therapeutic alliance) is not predictive of adolescents' eating disorder symptomatology or weight at the end of treatment (45). High parental expressed emotions have, however, been associated with a slower weight gain trajectory (38).

One interesting finding is that low levels of parental empowerment at entry into a FBT-informed DP predicted greater weight restoration at the end of treatment (53). While this finding may initially appear counterintuitive, one way to interpret this finding is that FBT-informed DPs are empowering and containing for parents.

#### Early Changes in Treatment and Outcome

Early change in three factors have been shown to predict improved outcomes in family-focused DPs; early weight gain, early cognitive change and early therapeutic alliance. Weight gain within the 1st month of DP treatment has been shown to predict discharge weight (31, 53). It has also been shown to predict remission defined broadly (47), although this was not replicated in a another study that used a more stringent remission criteria (31). With regard to eating disorder psychopathology, greater cognitive change within the 1st month (31) and stronger therapeutic alliance by week two (45) were both associated with end of treatment cognitive symptom improvement.

In the latter study, Rienecke et al. (45) note that early therapeutic alliance was also associated with lower symptom severity at admission, suggesting that this group may have had better outcomes because they were less severely unwell upon entry to their programme. Interestingly, therapeutic alliance with either parent did not predict improvements in eating disorder psychopathology or weight gain for the young people. Furthermore, therapeutic alliance appeared to form early (week 2) and did not significantly change over the rest of treatment for adolescents, mothers or fathers. Again, the first few weeks of treatment appear crucial. Early change has not been specifically investigated in all age programmes.

#### Therapeutic Model and Outcome

Two uncontrolled studies to date have directly examined whether the therapeutic model used within DP treatment impacts outcome (49, 61). After 3 years of operation, Huryk et al. (49) restructured their DP to be FBT-informed. They compared readmission rates to their programme before and after this change (*N* = 326) and found a significant reduction since the integration of FBT principles (11.7 vs. 2.9%). They also noted that since FBT was integrated, adolescents who attended their programme had a lower admission weight, had been ill for a shorter duration and were more likely to have anorexia nervosa as opposed to other types of eating disorder diagnoses.

Bean et al. (61) conducted a similar comparison on a much smaller sample (*N* = 16) of adolescents and young adults (12–20 years) with anorexia nervosa. They found that those who received FBT-informed DP treatment demonstrated significant improvements in weight, eating disorder symptoms and mood, whereas those who participated in a non-FBT informed DP only demonstrated weight improvements. While encouraging, this study is very small and the groups differed in clinical severity at baseline, which was not accounted for in statistical analyses.

One other study referred to potential improvements in outcomes due to a change in treatment model. In their discussion, Baudinet et al. (69) noted that after changing the therapeutic group programme from being predominantly informed by Dialectical Behavior Therapy (81) to Radically Open Dialectical Behavior Therapy (82) they had far fewer referrals to inpatient treatment (18 vs. 5%).

## Discussion

From this systematic scoping review of the adolescent eating disorder DP literature there are a few key findings that can be reported. Most commonly, studies are from North America (80%), report on programmes that operate 5-days-per-week (76%) and include predominantly adolescents or adolescents and young adults (84%) with restrictive eating disorders only (57%). Most studies have a model of treatment that is family focused (69%), although there is considerable variation in how much each programme adheres to one particular model vs. integrates multiple models. Even when a programme was described as being primarily informed by one treatment (e.g., FBT), it was common for other treatment modalities (e.g., CBT, DBT) to inform individual or group components of the treatment.

This review identified two main types of DPs currently operating. The first is typically for younger people only and informed by family-based treatment models. Typically, this type of DP is treating and/or exclusively reporting outcomes for underweight young people with restrictive eating disorder presentations. The second type of DP appears to be much more mixed with regard to age, type of presentation and the treatment modality, which appears to be more influenced by individual psychotherapy models with less or no emphasis on integrating family elements. Given the vastly different role the adolescents and parents play in the recovery process in these two types of programmes, it could be expected that the factors that will influence outcomes may vary depending on the type of programme. In the first type of programme parental factors may play a much bigger role in outcome and are potentially more modifiable given the level of parental involvement required during treatment. Conversely, individual factors such as illness severity, functional impairment and motivation may impact outcome more in the latter type of DP, as the onus of change and recovery is placed much more on the individual.

The only data directly comparing the impact of DP models on outcomes suggests being family-focused reduces DP readmission rates (49). It may also be associated with better weight and mood outcomes (61), although the latter findings are from a very small study (*N* = 16) with methodological limitations. Being more focused on specific personality predispositions associated with restrictive eating disorders may also reduce the need for inpatient treatment for this particular group (69). In addition, the largest study included in this review (*N* = 1,200) reported that younger people may have worse outcomes in all age, non-family focused DP treatment (37). This could suggest the need for age-, diagnosis- and model-specific DP treatments. However, not enough data is currently available to support or refute this. More data and direct comparisons of outcomes according to treatment model are needed.

An important consideration is also the impact of local healthcare and insurance systems. While insurance was only mentioned in one study, these systems will directly shape the admission and discharge criteria for all DPs, which population they target, the length of treatment and aims. The cost, availability and proximity of outpatient and inpatient treatment also needs to be considered, as it will directly influence the scope and length of DP treatment. If no other treatment is locally available or covered by insurance companies, programmes could potentially aim for full remission, rather than just clinical improvement. Eleven of the 13 studies that described weight targets for discharge were in North America, as were the three that report remission or partial remission rates. This suggests cultural and system differences in the aims and scope of DP treatment.

The use of a weight criterion at admission, as opposed to just medical stability, also highlights potential cultural and system differences in the scope and aims of DP treatment. This could differentiate those that act as a true alternative to inpatient treatment (for medical stabile adolescents), as opposed to being positioned as a higher intensity outpatient treatment (weight criterion). Further clarity and consistency in reporting of admission and discharge criteria, as well as healthcare system requirements, are needed to properly understand this.

Regarding outcomes, this review highlights DP treatment for adolescent eating disorders has non-inferior outcomes to inpatient care after brief stabilization (26) and may even be superior to inpatient treatment at longer term follow up (22). It is now relatively well-established that inpatient treatment beyond medical stabilization or containment of acute risk has limited benefit (13, 37). This review highlights that DP treatment is robustly associated with weight gain (for those who are underweight), reduced eating disorder symptoms, improvements in symptoms of comorbid depression and anxiety, as well as improvements in general functioning and quality of life. These improvements are generally maintained in the short- and medium-term, although some deterioration of symptoms, but not weight, is reported by some studies at 12-month follow-up.

It also appears that the initial few weeks of treatment are important for the treatment outcome. In family-focused adolescent and young adult only programmes early weight gain, early cognitive change, and therapeutic alliance within the first few weeks of treatment have been shown to predict outcome (31, 45, 47, 53). In all-age DPs, relatively fewer papers are published investigating predictors, moderators or mediators. Available data demonstrate that age, eating disorder diagnosis, motivation, symptoms of depression and worry at baseline have all been shown to influence outcomes by the end of treatment (37, 48, 73). Some studies report that eating disorder and comorbid symptom severity are associated with poorer outcomes, whereas others have not found these associations. The data are less clear regarding other individual factors and their association with outcome at discharge and follow-up.

Only 14 (29%) of the included studies report on parent/family factors. Interestingly, low parental empowerment at assessment was associated with better outcomes in one study (53). The additional support and intensity offered in a DP may help to instill hope in recovery and reactivate parents in ways that outpatient treatment might not be able to achieve. This may enable them to execute greater level of agency and effectiveness in their parental role. The way in which parental agency interacts with a relational containment of the adolescent, and how these factors impact outcome is yet to be fully understood. In the outpatient treatment context, relational containment is reportedly an important part of promoting recovery (83). It is possible that multi-disciplinary DP team offers relational containment to each family as a whole within the unique DP context. The findings on family factors highlight that multiple processes are occurring at the individual and family level in DPs, which require further exploration.

This will be important to consider in future research, particularly when examining more closely for whom DP treatment is appropriate and effective. There is a very limited data available regarding DP treatment response for young people with increased psychiatric complexity and risk e.g., trauma, abuse or neglect, emerging personality disorder traits, self-harm and those living in less typical family constellations, such as foster care or out-of-home care. Only three studies reported comorbid emerging personality disorder features within their sample and rates were very low (66, 68, 73). Furthermore, no study discusses considerations needed for those with trauma, abuse or more complex family circumstances. One study describe the careful considerations required for those with comorbid self-harm and increased risk (66).

The type of DP treatment may be particularly important to consider for this group of young people. The role of family involvement, when family relationships may be more complex and/or family supports limited, needs careful consideration. It is possible that the intensive relational containment offered within family-focused day programs may be beneficial for some, however, it may also be very unhelpful and distressing for others. In those circumstances, programmes informed by DBT may be more appropriate.

The mixed and sometime contradictory findings regarding predictors and mediators of DP treatment outcome are unsurprising in many ways. This review has shown that DPs have very different designs, treatment lengths and treatment philosophies. Furthermore, there is great variability in the quality of studies, sample size included and a marked dearth of controlled trials. The lack of consensus in defining outcome and recovery in the field of eating disorders generally complicates this matter even further (84). For all of these reasons, it is impossible to confidently compare the results of different adolescent DP studies. Rather, only trends can be highlighted.

Another key finding from the current review is that the physical and psychological aspects of recovery follow different trajectories in DP treatment (31, 48, 58). This suggests that the process and mechanism of change may also be different. Weight gain for those who are underweight is unsurprising in some ways, given it is often a compulsory requirement of DP treatment and contracted at assessment. Adolescents who do not gain weight are often quickly referred to higher levels of care or discharged. What is less clear, however, are the processes and mechanisms via which psychological change occurs. Most DPs offer a combination of several, often multi-model group-based, interventions that target specific psychological factors associated with eating disorders. The specific impact these interventions have on the psychological factors they are designed to target remains unknown. Furthermore, the way in which DP treatment model and structure, group process factors and family-focused interventions influence psychological (and physical) changes are also unknown. Investigating psychological factors and interventions that target them in future research is important given that both physical and psychological factors are essential for recovery (84) and may require different and specific treatment components.

Most studies reported a mean length of stay well below the typical outpatient treatment length of 6–12 months. The majority also state that the aim of DP treatment is clinical improvement, rather than remission. As such, the amount of expected change, particularly cognitive change, is likely to be modest, even in the most effective programmes. Behavioral change is often a precursor to cognitive change (85), and may be a sufficient treatment target for DP treatment, so long as it occurs within a continuum of care. Offering brief, intensive DP treatment followed by outpatient treatment may be the most appropriate model of care. It is likely to be the least restrictive and most cost-effective treatment pathway.

### Limitations

Several limitations are apparent from this review. Firstly, only English language and no gray literature was reviewed (conference abstracts, dissertations, etc.). Regarding the papers reviewed, most notable are the small sample sizes reported on, the uncontrolled nature of study methodologies and the lack of consistency in outcome reporting.

Only one RCT directly compared DP and inpatient treatment. Only two uncontrolled studies, one of which was very small, directly compared outcomes for different types of DP treatment. This makes it difficult to confidently say whether DPs do actually function as a true alternative to inpatient treatment.

With regard to sample size, 69% of the studies reviewed had sample sizes below 100 participants and 24% had 30 participants or less. This might suggest that many of the studies were underpowered, making the majority of conclusions very tentative. Several papers appear to be reporting different outcomes of roughly the same participant group, meaning the literature base may appear inflated compared to the actual current evidence base. Increased consistency in outcome reporting that includes independent effect sizes for both weight and cognitive-based AN symptomatology (86) and more detailed descriptions of the treatment models would also greatly improve the clarity of findings and comparability of studies. Additionally, consistency in how remission is defined and greater detail in reporting of what happens during follow up periods (e.g., details of ongoing treatment engagement and treatments received) is needed.

Lastly, from the current review, it is very hard to determine DP treatment response for young people with bulimia nervosa, binge eating disorder and other presentations not predominantly characterized by dietary restriction. The majority of data reports on outcomes for young people with restrictive eating disorders. Research focused on the aforementioned group would also clarify whether it is important to separate or mix diagnostic groups in treatment.

## Future Directions

The current review highlights several areas for future research into adolescent DPs for eating disorders. The voice of adolescents and parents is noticeably missing from the current literature. Similarly, therapeutic model and programme structure are both hypothesized to be important and powerful treatment mechanisms; however, few studies have directly investigated their direct impact on outcome.

Broadly, the field would benefit from:

1. Increased consistency in outcome reporting, including the inclusion of independent effect sizes for both physical and health markers of recovery.
2. Replication studies regarding the non-inferiority comparison of DP to inpatient treatment with respect to outcomes, patient and family satisfaction and costs.
3. Further investigation into whether certain individual or family factors indicate the appropriateness of DP over inpatient setting.
4. Controlled studies investigating whether specific DP's treatment content or therapeutic models lead to improved outcomes and for which group in regard to their age, diagnosis and family composition.
5. Qualitative investigations of DP treatment change processes and mechanisms.

Together these would help deepen our understanding of when and for whom DPs can be offered as an alternative to inpatient care. A subgroup of young people currently treated in inpatient units may not require such intensity. Understanding the characteristics of this subgroup and clarifying questions raised regarding DP treatment model, length and intensity will ensure all young people are treated in the least restrictive and most cost-effective ways possible.

## Author Contributions

JB and MS were involved in all aspects of this research. All authors contributed to the article and approved the submitted version.

## Conflict of Interest

The authors declare that the research was conducted in the absence of any commercial or financial relationships that could be construed as a potential conflict of interest.
