# Supplementary material for: Adolescent Eating Disorder Day Programme Treatment Models and Outcomes: A Systematic Scoping Review
Source: Front Psychiatry. 2021 Apr 29;12:652604. doi: 10.3389/fpsyt.2021.652604 (PMC8116630; doi:10.3389/fpsyt.2021.652604)
Supplement: Supplementary file 1 [file Data_Sheet_1.docx]

**Supplementary Material**

|  |
| --- |

**Coding Criteria for Day Program Treatment Models:**

***Family vs. Non-Family Focused***

*Primary Question:*

| **Question 1:** Primary therapeutic model defined/identified as a family-focused model (e.g. FBT, FT-AN) or non-family focussed model (e.g. CBT, psychodynamic, DBT)*  **Family focused / Non-family focussed / not specified (circle)**    *If specified, stop here as code identified*  *If not specified move to secondary questions below* |
| --- |

*Secondary Questions:*

| *#* | **Item** | **Y** | **N** | **Notes** |
| --- | --- | --- | --- | --- |
| *1* | Maudsley, FBT, FT-AN specified as a treatment model |  |  |  |
| *2* | Weekly family therapy offered |  |  |  |
| *3* | High level of parental involvement (every day) |  |  |  |
| *4* | Program is from a centre that has been defined as family focused (as above) in another peer-review publication. |  |  |  |
|  | **Total** |  |  |  |

| **Coding** |  |
| --- | --- |
| Family focussed | Identified as family focussed in Q1 *or* one or more of criteria #1-4 are marked as ‘yes’ |
| Non-family focused | Identified as non-family focussed in Q1 *or* criteria #1-4 are all marked ‘no’ |

**If two treatment models are identified as primary and one of them is family focussed (e.g. FBT and DBT), categorise as ‘family focused’*

Supplementary Table | Additional study characteristics

|  | ***Author*** | ***year*** | ***Population reported on*** | ***Family or non-family focussed*** | ***Comorbid diagnosis reported*** | ***Depression symptoms reported*** | ***Anxiety symptoms reported*** | ***Medication reported*** | ***Follow-up data reported*** | ***Data focus*** |
| --- | --- | --- | --- | --- | --- | --- | --- | --- | --- | --- |
| **USA** | Brown, et al. | 2020 | C&A | FF | N | N | N | N | Y | ind. |
|  | Parks, et al. | 2017 | C&A | FF | N | N | N | N | N | ind. |
|  | Rielly, et al. | 2019 | C&A | FF | Y | N | N | Y | N | ind. |
|  | Reilly, et al. | 2020 | YA | FF | Y | Y | Y | Y | Y | ind. |
|  | Freudenberg, et al. | 2016 | All | Non-FF | N | Y | N | N | N | ind. |
|  | Schaffner & Buchannan | 2008 | All | Non-FF | N | Y | Y | N | N | ind. |
|  | Schaffner & Buchannan | 2010 | All | Non-FF | N | Y | Y | N | N | ind. |
|  | Hayes, et al. | 2019 | All | Non-FF | Y | Y | N | N | N | ind. |
|  | Berona, et al. | 2018 | YA | FF | Y | N | N | N | N | ind.+parent |
|  | Homan, et al. | 2020 | YA | FF | N | Y | N | N | Y | ind.+parent |
|  | Hoste | 2015 | YA | FF | N | Y | N | N | N | ind.+parent |
|  | Rienecke | 2018 | C&A | FF | N | N | N | N | N | ind.+parent |
|  | Rienecke | 2019 | YA | FF | Y | N | N | N | N | ind.+parent |
|  | Rienecke | 2020 | C&A | FF | Y | Y | Y | Y | N | ind. |
|  | Rienecke & Ebeling | 2019 | C&A | FF | N | N | N | N | N | ind. |
|  | Rienecke & Richmond | 2018 | YA | FF | N | Y | N | N | Y | ind.+parent |
|  | Rienekce, et al. | 2016 | YA | FF | N | Y | Y | N | N | ind.+parent |
|  | Smith, et al. | 2019 | C&A | FF | N | N | Y | N | N | ind. |
|  | van Huysse, et al. | 2020 | C&A | FF | N | N | N | N | N | ind. |
|  | Fewell, et al. | 2017 | All | Non-FF | Y | Y | Y | N | Y | ind. |
|  | Huryk, et al. | 2020 | YA | FF | N | N | N | N | N | ind. |
|  | Dancyger, et al. | 2002 | C&A | Non-FF | Y | Y | N | Y | N | ind.+parent |
|  | Dancyger, et al. | 2003 | All | Non-FF | Y | Y | N | N | N | ind.+parent |
|  | deGraft-Johnson, et al. | 2013 | all | Non-FF | N | N | N | N | N | ind. |
|  | Wisotsky, et al. | 2003 | all | Non-FF | N | N | N | N | N | ind.+parent |
|  | Martin-Wagar, et al. | 2019 | C&A | FF | N | N | N | N | N | ind.+parent |
|  | Bustin et al. | 2013 | C&A | FF | Y | Y | Y | N | N | ind.+parent |
|  | Bryson, et al. | 2018 | C&A | FF | Y | N | N | Y | Y | ind. |
|  | Lane-Loney, et al. | 2020 | C&A | FF | N | Y | Y | N | N | ind.+parent |
|  | Nicely, et al. | 2014 | C&A | FF | Y | Y | Y | N | N | ind. |
|  | Ornstein, et al. | 2012 | C&A | FF | Y | Y | Y | Y | N | ind. |
|  | Ornstein, et al. | 2017 | C&A | FF | N | N | Y | Y | N | ind. |
|  | Zickgraf, et al. | 2019 | C&A | FF | Y | N | N | N | N | ind. |
|  | Bean, et al. | 2010 | YA | FF | N | Y | N | N | N | ind. |
| **CANADA** | Girz, et al. | 2013 | C&A | FF | Y | Y | Y | Y | N | ind.+parent |
|  | Grewal, et al., | 2014 | C&A | FF | Y | N | N | Y | N | ind. |
|  | Henderson, et al. | 2014 | C&A | FF | N | Y | Y | N | Y | ind. |
|  | Ngo & Isserlin | 2014 | C&A | Non-FF | N | N | N | N | N | ind. |
|  | Pennell, et al. | 2019 | C&A | FF | Y | N | N | N | N | ind. |
| **EUROPE** | Lazaro, et al. | 2010 | C&A | Non-FF | N | N | N | N | N | ind. |
|  | Serrano-Troncoso, et al. | 2020 | C&A | Non-FF | Y | N | N | Y | Y | ind. |
|  | Baudinet, et al. | 2020 | C&A | FF | Y | Y | N | N | N | ind. |
|  | Pretorius, et al. | 2015 | C&A | FF | N | N | N | N | N | ind. |
|  | Simic, et al. | 2018 | C&A | FF | N | Y | Y | N | Y | ind. |
|  | Herpertz-Dahlman, et al. | 2014 | C&A | Non-FF | Y | N | N | N | Y | ind. |
| **RoW** | Goldstein, et al. | 2010 | C&A | Non-FF | Y | N | N | Y | Y | ind. |
|  | Green, et al. | 2015 | YA | Non-FF | Y | Y | Y | Y | N | ind. |
|  | Danziger, et al. | 1988 | C&A | FF | N | N | N | N | Y | ind. |
|  | Danziger, et al. | 1989 | C&A | FF | N | N | N | N | Y | ind. |
|  |  |  | ***C&A: 31***  ***YA: 10***  ***All: 8*** | ***FF: 34***  ***Non-FF: 15*** | ***22*** | ***23*** | ***16*** | ***12*** | ***13*** | ***ind.: 35***  ***ind.+parent: 14*** |

*Abbreviations: C&A: child and adolescent; CAN: Canada; FF: family focusssed; ind.: individual; non-FF: non-family focussed; RoW: rest of world; USA: United States of America; YA: young adult.*
